# Accuracy of two geocoding methods for geographic information system-based exposure assessment in epidemiological studies

**DOI:** 10.1186/s12940-017-0217-5

**Published:** 2017-02-24

**Authors:** Elodie Faure, Aurélie M.N. Danjou, Françoise Clavel-Chapelon, Marie-Christine Boutron-Ruault, Laure Dossus, Béatrice Fervers

**Affiliations:** 1 Cancer and Environnent Department, Centre Léon Bérard, 28 rue Laennec, 69373, Lyon, Cedex 08 France; 2 Claude Bernard Lyon 1 University, 43 Boulevard du 11 Novembre 1918, 69100 Villeurbanne, France; 3 Inserm, Centre for research in Epidemiology and Population Health (CESP), U1018, Team “Generations for Health”, 94805 Villejuif, France; 4 Paris Sud University, UMRS 1018, 94805 Villejuif, France; 5 INSERM U1018 – EMT, Institut Gustave Roussy, 114 rue Edouard Vaillant, 94805 Villejuif, Cedex France

**Keywords:** Geocoding, Geographic information system, GIS, Epidemiology, Environmental epidemiology, Residential history

## Abstract

**Background:**

Environmental exposure assessment based on Geographic Information Systems (GIS) and study participants’ residential proximity to environmental exposure sources relies on the positional accuracy of subjects’ residences to avoid misclassification bias. Our study compared the positional accuracy of two automatic geocoding methods to a manual reference method.

**Methods:**

We geocoded 4,247 address records representing the residential history (1990–2008) of 1,685 women from the French national E3N cohort living in the Rhône-Alpes region. We compared two automatic geocoding methods, a free-online geocoding service (method A) and an in-house geocoder (method B), to a reference layer created by manually relocating addresses from method A (method R). For each automatic geocoding method, positional accuracy levels were compared according to the urban/rural status of addresses and time-periods (1990–2000, 2001–2008), using Chi Square tests. Kappa statistics were performed to assess agreement of positional accuracy of both methods A and B with the reference method, overall, by time-periods and by urban/rural status of addresses.

**Results:**

Respectively 81.4% and 84.4% of addresses were geocoded to the exact address (65.1% and 61.4%) or to the street segment (16.3% and 23.0%) with methods A and B. In the reference layer, geocoding accuracy was higher in urban areas compared to rural areas (74.4% vs. 10.5% addresses geocoded to the address or interpolated address level, *p* < 0.0001); no difference was observed according to the period of residence. Compared to the reference method, median positional errors were 0.0 m (IQR = 0.0-37.2 m) and 26.5 m (8.0-134.8 m), with positional errors <100 m for 82.5% and 71.3% of addresses, for method A and method B respectively. Positional agreement of method A and method B with method R was ‘substantial’ for both methods, with kappa coefficients of 0.60 and 0.61 for methods A and B, respectively.

**Conclusion:**

Our study demonstrates the feasibility of geocoding residential addresses in epidemiological studies not initially recorded for environmental exposure assessment, for both recent addresses and residence locations more than 20 years ago. Accuracy of the two automatic geocoding methods was comparable. The in-house method (B) allowed a better control of the geocoding process and was less time consuming.

## Background

Environmental epidemiology requires reliable assessment of both temporal and spatial components of exposure. In response to these challenges, epidemiological studies are increasingly using residential addresses of study participants and geographic information systems (GIS) to improve characterization of environmental exposures and examine their association with human health risks for a large variety of disease conditions [1]. GIS, for instance, have been used to investigate the relationship between environmental exposures and risk of breast cancer [2–4], leukemia [5–7], Parkinson’s diseases [8, 9], adverse birth outcomes [10, 11], and respiratory health [12–15]. GIS-based exposure assessment using residential proximity to the environmental exposure source (e.g. farmland treated with pesticides, industrial facilities or traffic roads) as an exposure surrogate relies on the positional accuracy of the subjects’ residences to avoid exposure misclassification [16]. There is increasing use of existing prospective cohorts for investigating environmental causes of diseases, although most of them had not been initially designed for environmental exposure assessment [17, 18]. While the strength of using existing cohorts relies on the prospective data collection at the individual level over many years allowing to adjust for individual disease risk factors, the subjects’ postal addresses have rarely been collected to be geocoded (i.e. to be converted into precise geographic coordinates) for their use in GIS. This may result in poor positional accuracy of subjects’ addresses and may represent an important source of misclassification and imprecision in environmental exposure assessment [13, 16, 19–24].

The process of geocoding and assigning geographic coordinates (latitude and longitude) to the study subject’s residential addresses is one of the first steps in GIS-based epidemiological studies [20, 24–26]. The quality of geocoding depends on the completeness and the level of positional accuracy of located addresses. Completeness is the proportion of addresses that can be geocoded and depends on the quality of the collected data on addresses. The positional accuracy reflects the level of proximity of geocoded objects to their true location [27, 28]. Geocoding residential addresses can be performed using three methods. A first method consists in using online geocoding services to obtain subjects’ coordinates or to create online maps with subjects’ residence locations [29, 30]. These free services are available on the Internet and do not require specific expertise in geocoding [21]. A second approach consists in using a commercial service that can handle all steps of geocoding from the spell checking of addresses to their map location [11, 13, 24, 31]. The third method is the use of an in-house method of geocoding where the geocoding process is handled by the research team using commercially available GIS software equipped with a geocoding tool and a reference street database [7, 21, 24, 32, 33]. In Europe, and particularly in France, there is a lack of studies comparing accuracy of geocoding between different geocoding tools, as well as according to characteristics of residential locations and date of residence.

Several American and European studies have evaluated the accuracy of different geocoding methods and of their reference network database in comparison to field location using Global Positioning System (GPS) [13, 20, 27, 34] and manual location based on aerial-photography [28, 35]. These studies have raised awareness on the divergence of geocoding accuracy between methods, with variations in median positional errors ranging from 25 m to 201 m. Also, accuracy levels of geocoding may vary according to the urban or rural status of the subjects’ residential location [20, 24, 35, 36]. Furthermore, studies investigating differences in geocoding accuracy of residential addresses by date of residence yielded inconsistent results [20, 36].

The few studies that have previously explored the feasibility and the quality of geocoding residential addresses of an existing cohort in a European context were conducted on small populations (i.e. *n* = 30 [29], *n* = 100 [27] or *n* = 354 [13]). Moreover, these studies did not explore the accuracy of geocoding over various geographical areas (urban or rural) or time periods. Furthermore, the spatial distribution characteristics of towns and rural settlements, street pattern (e.g. grid type, street lengths) and population density factors that have been shown to affect the accuracy of geocoding [20, 24, 35, 37], differ between Europe and the United States (where most of the previous studies were conducted). Our study aimed at comparing the accuracy of two automatic geocoding methods, an online method and an in-house method, with a manual method of geocoding used as the reference, in a French national prospective cohort initiated in 1990. The present study will assess the respective levels of accuracy and confidence of each geocoded method tested in the European context. Our study further assessed the geocoding accuracy according to urban and rural status of the addresses and the period of residence. The study was performed in order to subsequently use the most suitable method for geocoding of subjects’ residences to assess environmental exposure in a case–control study nested within the same prospective cohort with regard to positional accuracy, ethical use of addresses and privacy protection as well as time and resources required.

## Methods

### Study population

Our analysis involved study subjects from a nested case control study (the Geo3N research project) including 5,455 breast cancer cases and 5,455 matched controls that aimed at analyzing the association between environmental dioxin exposure and breast cancer risk in the E3N *(Etude Epidémiologique des Femmes de la Mutuelle Générale de l’Education Nationale)* cohort [38]. E3N is an ongoing prospective French cohort study of 98,995 women investigating female cancer risk factors. E3N is the French component of the European Prospective Investigation into Cancer and Nutrition [39]. E3N participants were enrolled in 1990 at the age of 40 to 65 years old and were members of a national teachers’ health insurance. Subjects are followed up by self-administered questionnaires every 2 to 3 years. E3N was approved by the French commission for Data Protection and Privacy. For the present analysis, we selected 1,730 subjects from the nested case control study. All selected subjects lived in the Rhône-Alpes region at recruitment in the E3N cohort. The Rhône-Alpes region covers a territory of 43,196 km^2^ with over 6 million inhabitants and presents a broad diversity of territories with rural, mountainous, and highly urbanized areas. Residential addresses of study participants were collected through the baseline and four follow-up questionnaires, sent in 1990, 1997, 2000, 2002, and 2005 respectively.

### Data cleaning

To improve standardization and quality of geocoding, all subjects’ addresses were verified manually for the spelling of street and municipality names, using free online databases referencing French postal codes (e.g. www.codespostaux.com/, www.pagesjaunes.fr/pagesblanches/). We also completed address fields of subjects (i.e. missing or incomplete postal code, municipality name, street name and street number) by matching with data from previous and subsequent questionnaires for similar residential location (e.g. same street name and same city name). After exclusion of 45 subjects with missing address, postal code, or municipality name, 1,685 subjects corresponding to a total of 4,247 addresses consecutively collected at each questionnaire between 1990 and 2008 were included in the analysis.

### Geocoding methods

**Fig. 1:**
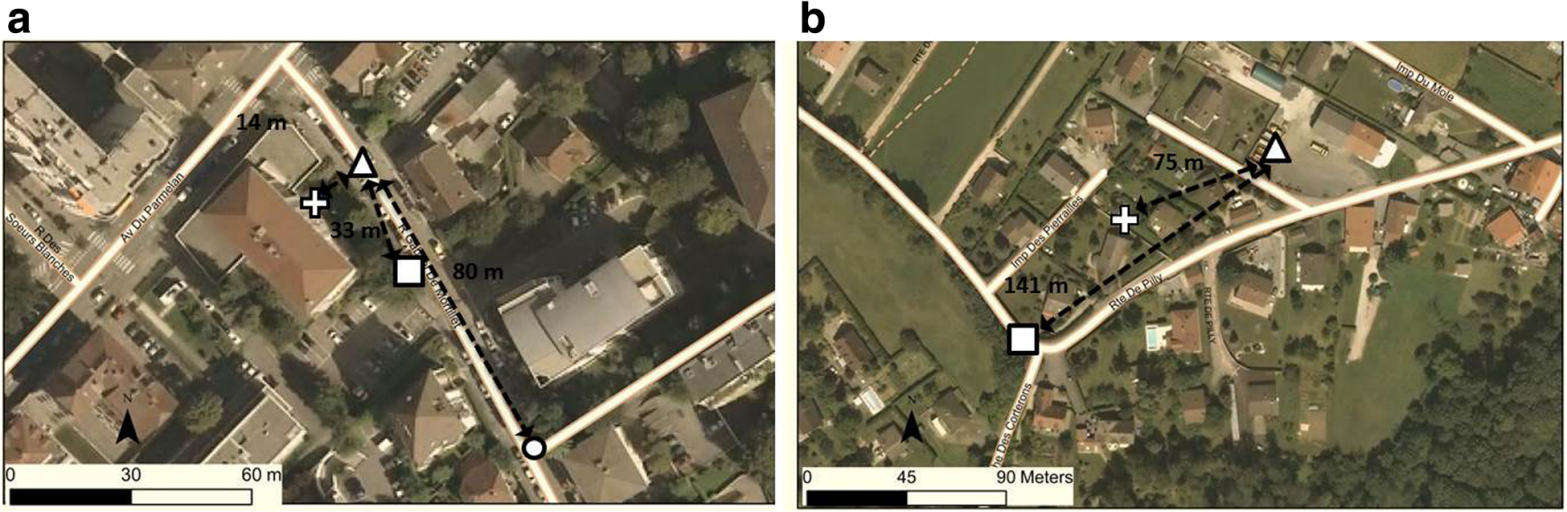
Illustration of address locations in urban and rural areas with the three distinct methods. **a** example of residence located in urban area; **b** example of residence located in rural area (circle: ArcGIS online location for method R (**a**); triangle: manually improved location with method R used as reference; cross: location with method A; square: location with method B; dashed lines representing the distances between addresses located with ArcGIS online for method R and method R (**a**); methods A and R (**a**, **b**) and methods B and R (**a**, **b**))

For each assessed method, dots representing addresses were located along the street at the entrance of the building (Fig. 1). A trained technician geocoded all addresses blinded to the case–control status of the subjects.

### Automatic methods

The first technique, “method A”, consisted in an automatic method based on a free online geocoding service accessible at http://dehaese.free.fr/Gmaps/testGeocoder.htm. The reference street network database was based on Google Maps^®^ ; the total number of addresses stored in the database was not provided by Google^®^. After automatic online geocoding processing, latitude and longitude coordinates in the WGS 84 projection system were exported for each address geocoded as well as accuracy of each location ranking from 0 to 9 (0: not found, 1: country level, 2: region (state and district), 3: county, 4: city, 5: postal code, 6: street segment, 7: intersection of streets, 8: address, 9: point of interest (building names, church…)). In France, levels 2, 3 and 7 did not exist in the administrative division of territories and were therefore not applicable. For geocoded addresses with a precision lower than 6 (street segment level), the spelling of street names and municipalities were checked again manually and corrected if necessary. Revised addresses were then geocoded a second time with the same online geocoder. The database was imported into ArcGIS 10.0 (Environmental System Research Institute- ESRI-Redlands, CA, USA) to create a data layer and all coordinates were converted into Lambert 93, which is the projection system currently used in France.

The second automatic method, “method B”, consisted in an in-house method based on the BD Adresse for ArcGIS [40, 41] and its reference street network database, BD Adresse® (National Geographic Institute, IGN, Saint Mandé, France) that includes 26 million addresses. For each geocoded address, the ArcGIS software provided two indicators to determine the best position: the spelling sensitivity that is the degree to which the spelling variation of a street name is allowed during a search for likely match candidates and the minimum candidate score that is a potential match record requires to be considered as a candidate [42]. The spelling sensitivity setting for an address locator is a value between 0 and 100. A higher value will restrict candidates to exact matches. Generally, the spelling sensitivity vary from 60 to 80 [21, 24, 43, 44], allowing only minor variations in spelling. Since the addresses in the cohort were not recorded to be geocoded, the correct spelling of street names or municipality was not certain. To maximize the proportion of participants and be assigned geographic coordinate, we used a lower spelling sensitivity, set arbitrarily to 50, based on the studies by Duncan et al. 2011 [21], Bell et al. 2012 [44] and Schootman et al. 2007 [43], to allow greater variation in spelling and retrieval of additional candidates. To select the most likely candidates with a high level of certainty without being too restrictive, we set the minimum candidate score to 80, similarly to previous studies conducted in the US [21, 24, 43, 44]. For method B, the geocoding accuracy levels ranked from 0 to 7: 0: not found, 1: exact address, 2: interpolated address, 3: street segment, 4: locality, 5: town hall, 6: postal code, 7: city. Interpolated addresses are located based on known positioned addresses along the street. For addresses with several possible matches (*N* = 499), we selected the address with the highest candidate score. Data were projected in Lambert 93.

### Reference method

The reference method in the present study, “method R”, was created by manually relocating addresses located by the online geocoder (http://dehaese.free.fr/Gmaps/testGeocoder.htm) used in method A and the ArcGIS online geocoding service. The location at the address level meant positioning of the address in front of the residence (house or building). As it was not feasible, timewise, to check manually all addresses, it was decided to check manually all addresses with an accuracy level equal to or lower than street segment (≤6) or all the addresses (accuracy level 8 or 9) deviating more than 50 m from the position obtained with an independent method of geocoding (ArcGIS online geocoding service). Considering property width, we considered locations accurate at 50 m. Consequently, all addresses with an accuracy level of 8 (address) or 9 (point of interest: school, hometown, etc.) were geocoded again with the ArcGIS online geocoding service [45]. The street network database of ArcGIS online is based on Navteq; the number of reference addresses stored in the database was not available from ESRI. For each address geocoded by both method A (batchgeocoder) and ArcGis online, the Euclidean distance between addresses geocoded by the online geocoder and the addresses geocoded by ArcGis Online was computed, using the point to point function in ArcGis 10.0. We used the Euclidean distance in the present study [13, 21, 24, 32, 46] as the (straight-line) distance from a person’s residence to an environmental exposure source has been shown to be a key factor of human exposure to environmental pollutants, such as dispersion of agricultural pesticides, dioxins or traffic-related emissions [5, 16, 47] and thus is used in GIS-based environmental exposure modelling. Addresses with a distance greater than 50 m were selected for manual checking and verified by a trained technician; dots were relocated to the right location when necessary. For manually checked addresses, relocation and the new accuracy level (town hall, locality, street segment or addresses) were recorded, as well as the information on the database used to determine manually the most accurate location (i.e. Google Maps®, Google Street View®, Geoportail®, Yahoo Map®, Geoportail® from IGN or BD Adresses® from IGN®) [35, 43, 48]. Addresses were located at the best available location based on the specification of the address itself (e.g., when the street number was missing, the best accuracy would have been to the street segment). When Geo3N addresses did not have exact postal address information (missing street name), the best location was the town hall. “Method R” was used as the reference to compare the accuracy of the two automatic geocoding methods (methods A and B).

### Data analyses

To facilitate comparison of the accuracy of the two automatic geocoding methods with the accuracy of method R, we regrouped the accuracy levels for each method into three categories, i.e. city or postal code, street segment, address or point of interest for method A; postal code or town hall, locality or street segment, interpolated address or address for method B; town hall, street segment or locality, address for method R. To assess the geocoding accuracy of each of the two automatic methods, we selected unique addresses among the 4,247 residential addresses collected consecutively. As the addresses were collected for each questionnaire, if the woman lived at the same address for two consecutive questionnaires, there were two addresses in the database. Minimal differences could occur in the spelling of these addresses. To identify unique addresses, we needed to know the points that overlap. Thus, we calculated X and Y coordinates of each automatically geocoded point from methods A and B, as well as the X and Y coordinates from method R. By matching X and Y coordinates from methods A and R, and methods B and R, we obtained respectively 2,224 and 2,425 pairs of X and Y coordinates corresponding to unique addresses. For method R, we obtained 2,112 unique pairs of X and Y coordinates. We computed two distance matrices, one for method A and one for method B, by calculating the Euclidean distance between each automatically geocoded unique address and its corresponding address in method R. We grouped distances into six categories (0–25 m, 26–50 m, 51–100 m, 101–400 m, 401–800 m and greater than 800 m) and the proportion of addresses in each category was computed for methods A and B. These categories were chosen to ease comparison of our results with those from previous studies [20, 24].

To provide details on the imprecision of addresses located at the street segment level, we calculated the median length of street segments (BD Adresses®from IGN) in urban and rural areas and in the city of Lyon. We selected a sample of streets located in the Rhône-Alpes region in a mainly urban department (Rhône) and a mainly rural department (Ardèche) to calculate, in each department, the median length of street segments overall and according to their rural or urban status. For each address, the urban or rural status was established for the year of residence using the French national institute for statistics and economic studies (INSEE) data. To account for changes in status over time, we used the 1990 urban area definition (UAD) for addresses from 1990 to 1995; the 1999 UAD for addresses from 1996 to 2004 and the 2010 UAD for addresses from 2005 to 2008. For each of the three geocoding methods, levels of accuracy were compared between urban and rural areas and according to two time-periods (1990–2000 and 2001–2008) using Chi-Square tests. All *p*-values were two-sided and *p*-values < 0.05 were considered as statistically significant. All *p*-values were two-sided and the significance level was set at 0.05. Cohen’s kappa coefficients were calculated to assess the agreement between the accuracies of methods A and B and the accuracy of method R [49]. The kappa coefficients were also calculated by time-periods (1990–2000 and 2000–2008) and urban/rural status. We used the SAS statistical software version 9.4 (SAS Institute Inc., Cary, North Carolina) for data analysis.

## Results

During the data cleaning step, the spelling of 235 addresses (5.5%) and 991 city names (23.3%) was corrected. Over the study period (1990–2008), more than 80% of the study population lived in urban areas and 75% of the study subjects remained at the same address throughout follow-up.

Based on 2,112 unique pairs of X and Y coordinates of addresses coded by method R, 723 (34.2%) addresses with an accuracy level lower or equal to six were checked manually and 329 (45.5%) of the latter were relocated. Also, 1,389 (65.8%) addresses with accuracy levels of 8 and 9 were re-geocoded with ArcGis online and 203 (14.6%) of the latter were relocated manually. Overall, with this reference layer, 63.2% of residence addresses were located to the address, 29.2% to the street segment or to the locality and 7.6% to the town hall. In the reference layer, 74.4% of addresses were located at the address level in urban areas versus 10.5% in rural areas (*p* < 0.0001, data not shown). The level of accuracy did not vary according to the time-period of residence: 62.1% and 63.3% of addresses were located at the address level for the time periods 1990–2000 and 2001–2008, respectively (*p* = 0.67, data not shown).

The positional errors of addresses located by method A and method B compared with method R are presented in Table 1. With method A, 405 (18.2%) addresses had a level of accuracy to the city or to the postal code, 363 (16.3%) to the street segment, 1,448 (65.1%) to the address or to the point of interest; 8 addresses (0.4%) could not be geocoded. Among addresses geocoded to the street segment, and address or point of interest, 226 (62.3%) and 1,241 (85.7%) respectively had a positional error of less than 25 m when compared with the layer generated by method R. For addresses geocoded to the city level or postal code, 160 (39.5%) had a positional error lower than 25 m and 146 (36.1%) above 400 m. Seventeen addresses (0.8%) had a positional error of over 30 km. The latter were incomplete and presented a wrong spelling of the municipality. Using method B, 1,490 (61.4%) of the addresses were geocoded to the point address or interpolated address level, 558 (23.0%) to the street segment or locality, and 377 (15.5%) to the town hall or postal code. One thousand (67.1%) addresses were located to the address or to interpolated address with a positional error of less than 25 m, as well as 53 (14.1%) were located at the postal code or town hall level, and 132 (23.7%) were located at the street segment. Addresses with the highest positional error (>400 m) compared with method R were geocoded to the town hall or the postal code (*n* = 244, 64.8%). Overall, 14.7% of addresses required manual checking with method B compared with 30.7% with method A, resulting in less geocoding time spent by the technician for method B.

**Table 1:** Comparison of positional errors^a^ (in meter) of addresses located by two automatic geocoding methods

Method of geocoding	Level of accuracy (accuracy code)	*N* addresses (%)	Median distance to the reference method location (IQR) in meters	Min-max, in meters	Distance in meters, *N* adresses (%)					
					[0–25]	[26–50]	[51–100]	[101–400]	[401–800]	>800
Method A^b^ (Batch Geocoder)	Not found (0)	8 (0.4)	5919.5 (815.4–5639076.5)	117.7–5679417.1	–	–	–	2 (25.0)	–	6 (75.0)
	City (4) or Postal code (5)	405 (18.2)	108.2 (0.0–787.3)	0.0–35204.7	160 (39.5)	13 (3.2)	26 (6.4)	60 (14.8)	48 (11.9)	98 (24.2)
	Street segment (6)	363 (16.3)	0.0 (0.0–108.1)	0.0–519630.8	226 (62.3)	18 (5.0)	25 (6.9)	41 (11.3)	10 (2.8)	43 (11.8)
	Address (8) or Point of interest (9)	1448 (65.1)	0.0 (0.0–0.0)	0.0–292249.4	1241 (85.7)	58 (4.0)	66 (4.6)	56 (3.9)	7 (0.5)	20 (1.4)
	Total	2224 (100.0)	0.0 (0.0–37.2)	0.0–5679417.1	1627 (73.2)	89 (4.0)	117 (5.3)	159 (7.1)	65 (2.9)	167 (7.5)
Method B (ArcGis Locator)	Postal code (6) or Town Hall (5)	377 (15.5)	788.9 (114.0–1845.1)	0.3–8545.1	53 (14.1)	11 (2.9)	25 (6.6)	44 (11.7)	56 (14.9)	188 (49.9)
	Locality (4) or Street segment (3)	558 (23.0)	110.3 (26.7–320.6)	0.0–14477.6	132 (23.7)	55 (9.9)	82 (14.7)	174 (31.2)	47 (8.4)	68 (12.2)
	Interpolated address (2) or Address (1)	1490 (61.4)	12.5 (6.0–35.6)	0.0–3951.9	1000 (67.1)	237 (15.9)	133 (8.9)	100 (6.7)	5 (0.3)	15 (1.0)
	Total	2425 (100.0)	26.5 (8.0–134.8)	0.0–14477.6	1185 (48.9)	303 (12.5)	240 (9.9)	318 (13.1)	108 (4.5)	271 (11.2)

^a^positional error was determined by calculating Euclide an distance (in meter) between addresses located by each automatic method (method A and method B) of geocoding regarding to a reference method (method R)

^b^the administrative division of territories in France did not allow obtaining addresses geocoded to district and state levels (level 2), county (level 3) and intersection of streets (level 7). No addresses have been geocoded to the country level

The median length of street segments in urban areas was 265 m in the Rhône department (223 m for the city of Lyon) and 209 m in Ardèche, while the median lengths of streets segments in rural areas was 252 m in Rhône and 411 m in Ardèche.

The concordance with method R was assessed separately for each of the two automatic methods (Table 2). Overall, Kappa coefficients were 0.60 between methods A and R and 0.61 between methods B and R. For addresses located in urban areas agreements of 0.56 and 0.52 were found respectively between methods A and R, and methods B and R, while in rural areas, agreements with method R were 0.39 and 0.54 for methods A and B respectively. Agreement with method R was 0.61 and 0.60 respectively for methods A and B for the period 1990–1999 and 0.56 and 0.70 respectively for methods A and B for the period 2000–2008.

**Table 2:** Agreement in accuracy level (Cohen’s Kappa coefficient) for methods A and B (automatic geocoding) in comparison with method R (manual reference method)

	Method R	Method A					Method B				
		City (N addresses)	Street segment (N addresses)	Address (N addresses)	Total (N addresses)	Cohen's kappa coefficient	Postal code (N addresses)	Street segment (N addresses)	Address (N addresses)	Total (N addresses)	Cohen's kappa coefficient
Overall	Town Hall	147	20	14	181	0.60	133	49	5	187	0.61
	Street segment	212	309	140	661		119	439	131	689	
	Address	46	34	1294	1374		125	70	1354	1549	
	Total	405	363	1448	2216		377	558	1490	2425	
For urban addresses^a^	Town Hall	62	10	11	83	0.56	54	27	5	86	0.52
	Street segment	94	166	130	390		64	226	126	416	
	Address	44	26	1265	1335		114	63	1331	1508	
	Total	200	202	1406	1808		232	316	1462	2010	
For rural addresses^a^	Town Hall	85	10	3	98	0.39	79	22	0	101	0.54
	Street segment	118	143	10	271		55	213	5	273	
	Address	2	8	29	39		11	7	23	41	
	Total	205	161	42	408		145	242	28	415	
For 1990–2000 period addresses	Town Hall	133	17	13	163	0.61	125	45	5	175	0.60
	Street segment	182	278	120	580		112	396	117	625	
	Address	45	29	1141	1215		121	67	1216	1404	
	Total	360	324	1274	1958		358	508	1338	2204	
For 2000–2008 period addresses	Town Hall	14	3	1	18	0.56	8	4	0	12	0.70
	Street segment	30	31	20	81		7	43	14	64	
	Address	1	5	153	159		4	3	138	145	
	Total	45	39	174	258		19	50	152	221	

^a^Assignment of urban and rural status of addresses was based on definitions established by the French national institute for statistics and economic studies (INSEE)

## Discussion

In the present study, we compared the accuracy of two automatic geocoding methods, overall, and according to urban or rural status of addresses and to the time period of residence (from 1990 to 2008). Compared with the reference method, the two methods of geocoding gave similar results in terms of general accuracy, with more than 60% of addresses geocoded to the exact address level, and more than 15% to the street segment level. Accuracy was higher in urban areas than in rural areas, while no difference was observed according to the period of residence. Compared with method A, method B allowed more control at all steps of the geocoding process and was less time consuming, in particular regarding the manual checking.

Based on the Euclidean distance to the address located by method R, 82.5% of addresses geocoded with method A and 71.3% of addresses with method B had a positional error lower than 100 m. This difference can be explained by the use of the same initial automatic geocoding (online geocoding) for method A and method R. Overall, these proportions are comparable to those from other studies conducted in France and outside France, with figures of 80.9% to 82.0% of addresses with positional error below 100 m in two French studies [31, 36], and 72.0% to 86.0% in international studies [20, 24, 34]. The accuracy level may have important implications on misclassification of individuals’ exposure, depending on the spatial concentration gradient of the exposure of interest and should be considered in the study design. While these geocoding errors observed in our study appear overall modest in magnitude, Ganguly et al. showed that positional errors exceeding 100 m may alter exposure estimates, in particular for exposures with important spatial gradients, such as traffic-related air pollution [16, 50]. To minimize misclassification, studies investigating this type of exposure should include only addresses with a level of accuracy at the address level and exclude those with an accuracy level at the street segment or the locality. This would be even more important for studies investigating the health impact of high voltage showing an even steeper spatial gradient [31, 51]. For other exposures, such as airborne dioxins emitted by industries with elevated stack height, the pollutant concentrations decrease to near background levels at distances of 3 km to 5 km making it possible to include geocoded addresses both at the street segment and at the locality levels [52, 53]. These observations stress the importance of conducting sensitivity analyses to examine the potential impact of positional errors on exposure estimates.

Our findings are consistent with other studies showing a more precise and accurate geocoding for addresses located in urban areas compared to rural areas [20, 24, 35, 36]. In these studies, the median values of the positional error ranked from 31 m to 56 m in urban areas and from 45 m to 212 m in rural areas where addresses lack frequently street number and are often limited to the name of the hamlet. Three studies (two US and one French) have geocoded historical addresses covering periods ranking respectively from 1948 to 2000, 1930 to 2000, and 1960 to 2001 [2, 20, 36]. In agreement with the present study, two of them did not observe major variations in the positional accuracy according to the timing of addresses [20, 36], whereas Brody et al., a US study, reported a better positional accuracy for recent addresses, with 37% of addresses located to the address level in 1930 vs. 62% for 1970–1980 and 97% for 1990–2001 [2]. Kappa coefficients showed overall good agreement with the reference method for the two automatic methods. However, for rural addresses, agreement was higher with method B compared with method A (0.54 vs. 0.39, respectively). Also, recent addresses (from 2000–2008) showed a higher Kappa coefficient for method B compared with method A (0.70 vs. 0.56).

**Fig. 2:**
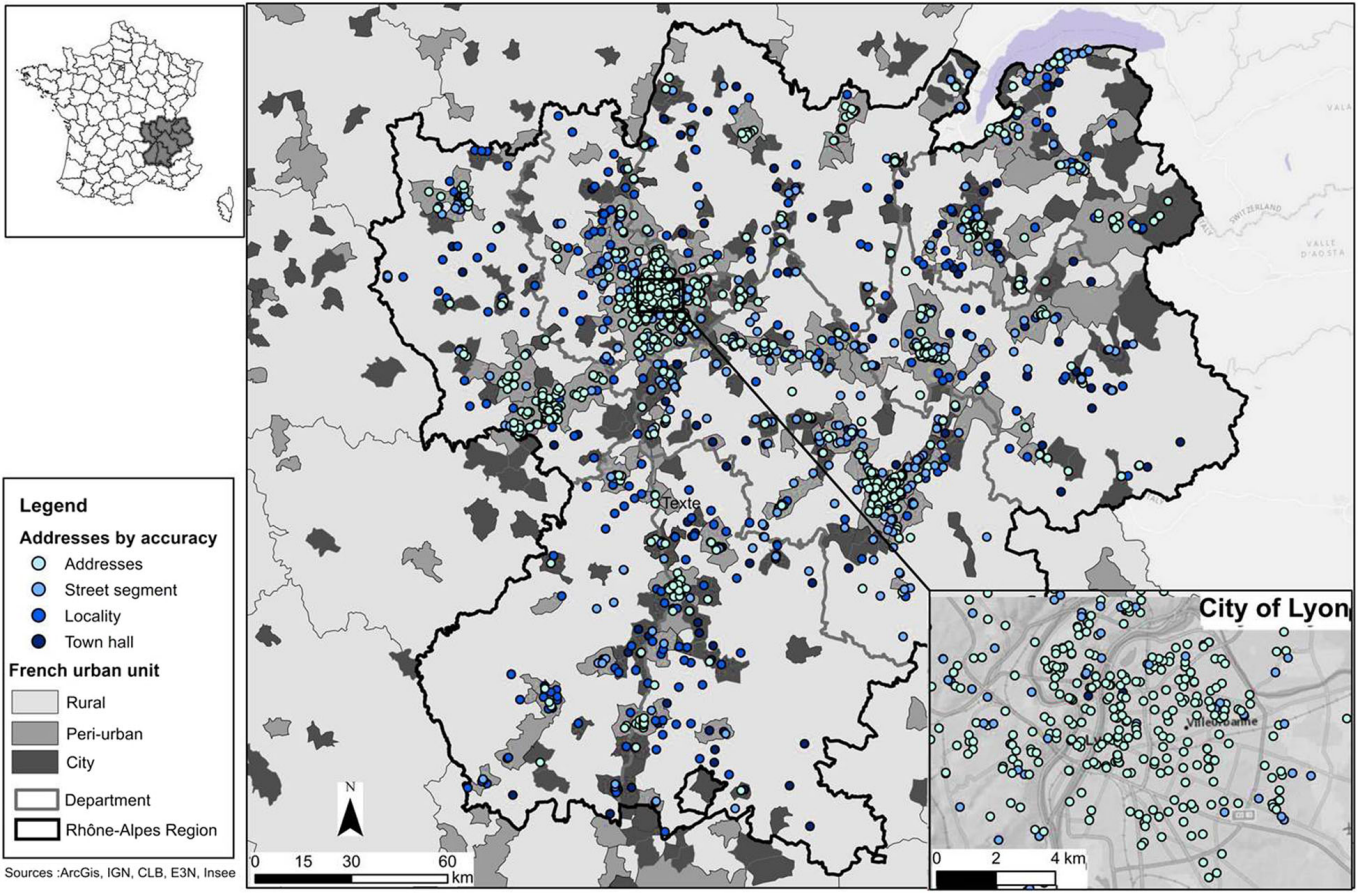
Accuracy level of addresses (located with method R) of the study population and their distribution according to urban unit in the Rhône-Alpes region and the city of Lyon

The strengths of our study include the large number of addresses (*n* = 4,247) and study subjects (*n* = 1,685) for whom residential addresses had been prospectively recorded over a 19-year study period (1900–2008). Moreover, we were able to classify addresses according to the rural or urban status of the area of residence. Our study is one of the first to investigate the geocoding of subjects from a national prospective cohort and offer both a spatial and temporal analysis on the quality of geocoding using different tools. In addition, the manual checking of the correct location of addresses was done for a large number of addresses and based on aerial images (Fig. 2). However, our study has several limitations. First, E3N addresses were not initially designed to be geocoded and this could have affected positional accuracy. However, the findings on addresses located with a positional error lower than 100 m were consistent with another French study in which addresses were recorded to be geocoded [36]. Also, to take into account potential errors in the spelling of addresses not collected to be geocoded we used, for method B, a threshold of 50 for spelling sensitivity. A higher threshold, as used by some authors, would have allowed only minor spelling variations of addresses and restrict the candidates to exact matches [21, 24, 41, 43]. As E3N participants are mostly teachers, some of them indicated only the name of their school (*n* = 49) or workplace (*n* = 6) in the address field; because these names are not available as such in reference databases, those could not be automatically geocoded and this may have had a minor impact on the global accuracy of both automatic geocoding methods. Second, because of the large number of addresses in our study and size of the study territory, as well as bad GPS signal reception in cities [54] feasibility of using field GPS location to validate the true location of all addresses, as performed by others was limited [13, 20, 24, 34]. However, the use of aerial photography [28, 44, 55] via Google Maps^®^, Geoportail^®^ and Google Street View® to manually check all addresses initially geocoded with a low level of accuracy, allowed us to be confident in the precision of the address location in our reference layer (method R). Third, one weakness of methods A and R is the impossibility, despite repeatedly contacting Google and ESRI France, to obtain the number of addresses recorded in their reference database.

Furthermore, geocoding of addresses in environmental epidemiology using external services or free online devices, such as the batch online geocoding, raises privacy and ethical considerations [56]. Since addresses may allow the personal identification of the study subjects, their transfer to third parties may breach participants’ confidentiality and anonymity, even after removal of any sensitive information, and in particular in defined geographic areas with small numbers of study subjects. In-house geocoding generally allows a better control of any type of unauthorized access to sensitive information.

The present study confirmed the geocoding method to be used in the E3N national cohort as a basis of GIS-based exposure modelling of environmental pollutants at the national level and analyses of related disease risk, such as breast cancer. The findings will contribute to strengthen the reliability of geocoding/GIS-based methods to assess environmental exposures, while taking into account privacy and ethical issues. Our results can be further used for applications in other European cohorts to make greater and more efficient use of the impressive resource of existing cohort data, to investigate environmental risk factors based on past and current places of residence. Thus, the present study could be reproduced in other European cohorts by integrating national road network databases into GIS software (i.e. ARCGIS). Future studies should precisely explore the impact of positional errors and accuracy level of addresses on misclassification for various environmental pollutants with varying distance decline pattern. Further methodological work is still needed on the feasibility of precisely geocoding addresses before 1990 (complete residential history from birth to recruitment into the cohort) in order to assess lifetime exposures from birth onward.

## Conclusion

Our study demonstrated the feasibility of geocoding addresses in epidemiological studies not initially designed to be used for environmental exposure assessment purposes, for both recent addresses and residence locations dated from more than 20 years. Furthermore, our results showed no major difference in final geocoding accuracy between the two automatic geocoding methods, compared with the manual reference method. Overall, more addresses showed a positional error lower than 100 m with method A, while the Kappa coefficients showed higher agreement with the reference method for method B, for both rural areas and the 2000–2008 period. Also, this in-house method allowed a better control at all steps of the geocoding process and was less time consuming. Future epidemiological studies should prospectively record residential addresses in a way that would improve geocoding for environmental exposure assessment. Finally, knowing the accuracy of the geocoding tool used in the context of environmental exposure assessment will help to limit misclassification bias due to positional errors. Epidemiological studies should be able to report their street network reference database and the accuracy of their geocoding method.

## Abbreviations

ESRI: Environmental systems research institute
GIS: Geographical information system
GPS: Global positioning system
IGN: National geographic institute
INSEE: French national institute for statistics and economic studies
UAD: Urban area definition
WGS: World geodetic system

## References

[1] Han D, Bonner MR, Nie J, Freudenheim JL (2013). Assessing bias associated with geocoding of historical residence in epidemiology research. Geospat Health.

[2] Brody JG, Aschengrau A, McKelvey W, Rudel RA, Swartz CH, Kennedy T (2004). Breast cancer risk and historical exposure to pesticides from wide-area applications assessed with GIS. Environ Health Perspect.

[3] O’Leary ES, Vena JE, Freudenheim JL, Brasure J (2004). Pesticide exposure and risk of breast cancer: a nested case–control study of residentially stable women living on Long Island. Environ Res.

[4] Reynolds P, Hurley SE, Gunier RB, Yerabati S, Quach T, Hertz A (2005). Residential proximity to agricultural pesticide use and incidence of breast cancer in California, 1988–1997. Environ Health Perspect.

[5] Gunier RB, Ward MH, Airola M, Bell EM, Colt J, Nishioka M (2011). Determinants of agricultural pesticide concentrations in carpet dust. Environ Health Perspect.

[6] Hoffmann W, Terschüeren C, Heimpel H, Feller A, Butte W, Hostrup O (2008). Population-based research on occupational and environmental factors for leukemia and non-Hodgkin’s lymphoma: the northern Germany leukemia and lymphoma study (NLL). Am J Ind Med.

[7] Rull RP, Gunier R, Von Behren J, Hertz A, Crouse V, Buffler PA (2009). Residential proximity to agricultural pesticide applications and childhood acute lymphoblastic leukemia. Environ Res.

[8] Lee P-C, Bordelon Y, Bronstein J, Ritz B (2012). Traumatic brain injury, paraquat exposure, and their relationship to Parkinson disease. Neurology.

[9] Ritz B, Costello S (2006). Geographic model and biomarker-derived measures of pesticide exposure and Parkinson’s disease. Ann N Y Acad Sci.

[10] Boccolini Pde MM, Boccolini CS, Meyer A, Chrisman Jde R, Guimarães RM, Veríssimo G (2013). Pesticide exposure and low birth weight prevalence in Brazil. Int J Hyg Environ Health.

[11] Chevrier C, Serrano T, Lecerf R, Limon G, Petit C, Monfort C (2014). Environmental determinants of the urinary concentrations of herbicides during pregnancy: the PELAGIE mother-child cohort (France). Environ Int.

[12] Gorai AK, Tuluri F, Tchounwou PB (2014). A GIS based approach for assessing the association between air pollution and asthma in New York State, USA. Int J Environ Res Public Health.

[13] Jacquemin B, Lepeule J, Boudier A, Arnould C, Benmerad M, Chappaz C (2013). Impact of geocoding methods on associations between long-term exposure to urban air pollution and lung function. Environ Health Perspect.

[14] Nuvolone D, Barchielli A, Forastiere F, Gruppo collaborativo EpiAir (2009). Traffic patterns regulations to reduce air pollution in the Italian cities participating in the EpiAir Project. Epidemiol Prev.

[15] Ranzi A, Porta D, Badaloni C, Cesaroni G, Lauriola P, Davoli M (2014). Exposure to air pollution and respiratory symptoms during the first 7 years of life in an Italian birth cohort. Occup Environ Med.

[16] Ganguly R, Batterman S, Isakov V, Snyder M, Breen M, Brakefield-Caldwell W (2015). Effect of geocoding errors on traffic-related air pollutant exposure and concentration estimates. J Expo Sci Environ Epidemiol.

[17] Beelen R, Hoek G, Raaschou-Nielsen O, Stafoggia M, Andersen ZJ, Weinmayr G, et al. Natural Cause Mortality and Long-Term Exposure to Particle Components: An Analysis of 19 European Cohorts within the Multi-Center ESCAPE Project. Environ Health Perspect. 2015 [cited 2016 Aug 10]; Available from: http://ehp.niehs.nih.gov/1408095. Accessed 20 Mar 2016. 10.1289/ehp.1408095 PMC4455583 25712504

[18] Jacquemin B, Siroux V, Sanchez M, Carsin A-E, Schikowski T, Adam M (2015). Ambient air pollution and adult asthma incidence in six European cohorts (ESCAPE). Environ Health Perspect.

[19] Zandbergen PA, Green JW (2007). Error and bias in determining exposure potential of children at school locations using proximity-based GIS techniques. Environ Health Perspect.

[20] Bonner MR, Han D, Nie J, Rogerson P, Vena JE, Freudenheim JL (2003). Positional accuracy of geocoded addresses in epidemiologic research. Epidemiol Camb Mass.

[21] Duncan DT, Castro MC, Blossom JC, Bennett GG, Gortmaker SL (2011). Evaluation of the positional difference between two common geocoding methods. Geospat Health.

[22] Jacquez GM (2012). A research agenda: does geocoding positional error matter in health GIS studies?. Spat SpatioTemporal Epidemiol.

[23] Kravets N, Hadden WC (2007). The accuracy of address coding and the effects of coding errors. Health Place.

[24] Ward MH, Nuckols JR, Giglierano J, Bonner MR, Wolter C, Airola M (2005). Positional accuracy of two methods of geocoding. Epidemiol Camb Mass.

[25] Rushton G, Armstrong MP, Gittler J, Greene BR, Pavlik CE, West MM (2006). Geocoding in cancer research: a review. Am J Prev Med.

[26] Zhang Z, Manjourides J, Cohen T, Hu Y, Jiang Q (2016). Spatial measurement errors in the field of spatial epidemiology. Int J Health Geogr.

[27] Ribeiro AI, Olhero A, Teixeira H, Magalhães A, Pina MF (2014). Tools for address georeferencing - limitations and opportunities every public health professional should be aware of. PLoS One.

[28] Zandbergen PA (2007). Influence of geocoding quality on environmental exposure assessment of children living near high traffic roads. BMC Public Health.

[29] Quesada JA, Nolasco A, Moncho J (2013). [Comparison of Google and yahoo applications for geocoding of postal addresses in epidemiological studies]. Rev Esp Salud Publica.

[30] Nuvolone D, Santini M, Pepe P, Cipriani F (2016). [Impacts of geocoding quality in environmental epidemiology studies: two case-studies in Tuscany Region (Central Italy)]. Epidemiol Prev.

[31] Sermage-Faure C, Demoury C, Rudant J, Goujon-Bellec S, Guyot-Goubin A, Deschamps F (2013). Childhood leukaemia close to high-voltage power lines—the geocap study, 2002–2007. Br J Cancer.

[32] Jones RR, DellaValle CT, Flory AR, Nordan A, Hoppin JA, Hofmann JN (2014). Accuracy of residential geocoding in the agricultural health study. Int J Health Geogr.

[33] Baldovin T, Zangrando D, Casale P, Ferrarese F, Bertoncello C, Buja A (2015). Geocoding health data with geographic information systems: a pilot study in northeast Italy for developing a standardized data-acquiring format. J Prev Med Hyg.

[34] Zhan FB, Brender JD, De Lima I, Suarez L, Langlois PH (2006). Match rate and positional accuracy of two geocoding methods for epidemiologic research. Ann Epidemiol.

[35] Cayo MR, Talbot TO (2003). Positional error in automated geocoding of residential addresses. Int J Health Geogr.

[36] Béranger R, Blain J, Baudinet C, Faure É, Fléchon A, Boyle H (2014). Tumeurs germinales du testiculeet expositions précoces aux pesticides: étude pilote TESTEPERA. Bull Cancer (Paris).

[37] Sonderman JS, Mumma MT, Cohen SS, Cope EL, Blot WJ, Signorello LB (2012). A multi-stage approach to maximizing geocoding success in a large population-based cohort study through automated and interactive processes. Geospat Health.

[38] Clavel-Chapelon F, E3N Study Group (2015). Cohort profile: the french E3N cohort study. Int J Epidemiol.

[39] Riboli E, Hunt K, Slimani N, Ferrari P, Norat T, Fahey M (2002). European prospective investigation into cancer and nutrition (EPIC): study populations and data collection. Public Health Nutr.

[40] ESRI France. BD Adresse for ArcGis. 2015 [cited 2015 Oct 26]. Available from: https://www.esrifrance.fr/iso_album/SPECS_BDADRESSE.pdf. Accessed 26 Oct 2015.

[41] ESRI. ArcGIS Help 10.1 - Geocoding option properties. 2015 [cited 2015 Oct 26]. Available from: http://resources.arcgis.com/en/help/main/10.1/. Accessed 26 Oct 2015.

[42] ESRI. Esri Support GIS Dictionary. 2015 [cited 2016 Sep 22]. Available from: http://support.esri.com/other-resources/gis-dictionary/browse/m. Accessed 22 Sept 2016.

[43] Schootman M, Sterling DA, Struthers J, Yan Y, Laboube T, Emo B (2007). Positional accuracy and geographic bias of four methods of geocoding in epidemiologic research. Ann Epidemiol.

[44] Bell S, Wilson K, Shah TI, Gersher S, Elliott T (2012). Investigating impacts of positional error on potential health care accessibility. Spat SpatioTemporal Epidemiol.

[45] ESRI. Geocode coverage. 2015 [cited 2015 Oct 26]. Available from: https://doc.arcgis.com/en/arcgis-online/reference/geocode-coverage.htm. Accessed 1 Sept 2015.

[46] Mazumdar S, Rushton G, Smith BJ, Zimmerman DL, Donham KJ (2008). Geocoding accuracy and the recovery of relationships between environmental exposures and health. Int J Health Geogr.

[47] Brody JG, Vorhees DJ, Melly SJ, Swedis SR, Drivas PJ, Rudel RA (2002). Using GIS and historical records to reconstruct residential exposure to large-scale pesticide application. J Expo Anal Environ Epidemiol.

[48] IGN IN de l’Information G et F. Le portail des territoires et des citoyens - Géoportail. 2015 [cited 2015 Oct 26]. Available from: http://www.geoportail.gouv.fr/accueil. Accessed 20 Oct 2015.

[49] Cohen J (1960). A coefficient of agreement for nominal scales. Educ Psychol Meas.

[50] Houot J, Marquant F, Goujon S, Faure L, Honoré C, Roth M-H (2015). Residential proximity to heavy-traffic roads, benzene exposure, and childhood leukemia—the GEOCAP study, 2002–2007. Am J Epidemiol.

[51] Draper G, Vincent T, Kroll ME, Swanson J (2005). Childhood cancer in relation to distance from high voltage power lines in England and wales: a case–control study. BMJ.

[52] Fabre P, Daniau C, Goria S, de Crouy-Chanel P, Empereur-Bissonnet P. Étude d’incidence des cancers à proximité des usines d’incinération d’ordures ménagères. Synthèse St.-Maurice InVS. 2008 [cited 2015 Aug 21]; Available from: http://opac.invs.sante.fr/doc_num.php?explnum_id=3306. Accessed 10 Jan 2014.

[53] Fréry N, Zeghnoun A, Sarter H, Falq G, Pascal M, Bérat B, et al. Etude d’imprégnation par les dioxines des populations vivant à proximité d’usines d’incinération d’ordures ménagères - Rapport d’étude. Institut de veille sanitaire. Saint-Maurine (Fra); 2009. Available from: http://opac.invs.sante.fr/doc_num.php?explnum_id=682. Accessed 10 Jan 2014.

[54] Hsu L-T, Gu Y, Kamijo S (2015). NLOS Correction/Exclusion for GNSS Measurement Using RAIM and City Building Models. Sensors.

[55] Matthew Bucklew, Caitlin Curry, Noah Krach, Jessica Molnar, Robert Stancil, John M Tilghman, et al. Undergraduate Research in Action: Evaluating the positional differences between the Google Maps and the United States Census Bureau geocoding APIs. 2016 [cited 2017 Jan 10]; Available from: https://doi.org/10.13140/RG.2.2.10457.93287. Accessed 29 Dec 2016.

[56] Mazumdar S, Konings P, Hewett M, Bagheri N, McRae I, Del Fante P (2014). Protecting the privacy of individual general practice patient electronic records for geospatial epidemiology research. Aust N Z J Public Health.

